# Examination of dietary habits among the indigenous Kuna Indians of Panama

**DOI:** 10.1186/s12937-019-0469-8

**Published:** 2019-08-01

**Authors:** Allison L. Neitzel, Brittany L. Smalls, Rebekah J. Walker, Aprill Z. Dawson, Jennifer A. Campbell, Leonard E. Egede

**Affiliations:** 1 College of Medicine, Medical College of Wisconsin, 8701 W Watertown Plank Road, Milwaukee, WI 53226 USA; 2 Center for Health Services Research, Department of Internal Medicine, University of Kentucky, 740 S Limestone Street, Lexington, KY 40536 USA; 3 Division of General Internal Medicine, Department of Medicine, Medical College of Wisconsin, 9200 W Wisconsin Ave, Milwaukee, WI 53226 USA; 4 Center for Advancing Population Science, Medical College of Wisconsin, 8701 Watertown Plank Road, Milwaukee, WI 53226 USA

**Keywords:** Indigenous population, Food consumption, Nutrition, Dietary patterns

## Abstract

**Background:**

Evidence for dietary habits among the Kuna Indians of Panama outside of cacao consumption is limited. Global trends suggest an uptake in processed foods conferring risk for chronic disease. This paper aims to provide information on dietary habits and investigate sociodemographic correlates of diet for the indigenous population living off the coast of Panama.

**Methods:**

This sample included 211 Kuna Indians ages 18 years or older living within the island communities of Ustupu and Ogobsucum. Cross-sectional data was collected using a paper-based survey to assess dietary patterns. Categories of food included: fruits, vegetables, cacao, fish, sodas, fried, junk, and fast foods. Univariate analyses were used to describe demographic variables, followed by chi-squared tests to understand individual correlates of food types.

**Results:**

About 85% reported eating fast food at least weekly, 47% reported eating fried food daily, and 11% reported eating junk food daily. Forty-three percent of the sample population reported eating fish daily. Those with poor incomes reported more fish consumption than any other income group (51%, *p* = 0.02). After adjusting for all covariates, those in higher income categories were less likely to eat fruits, cacao, and fish daily, but were also less likely to eat fast food weekly and junk food daily. Elderly populations (age 60–90 OR = 12.17, 95%CI 2.00, 73.84), women (OR = 3.43, 95%CI 1.23, 9.56), and those with primary education (OR = 4.83, 95%CI 1.01, 23.0) were also more likely to eat fast food weekly.

**Conclusion:**

This is the first dietary survey study of the Kuna that focuses on food groups outside of cacao. Results suggest the community could benefit from efforts to increase cultivation of fruits and vegetables and reduce the percentage of energy consumption contributed by fast food, fried food, and junk food.

**Trial registration:**

N/A

## Introduction

The world’s indigenous population exceeds 370 million and is represented across 70 countries on all occupied continents [1, 2]. Globally, indigenous populations comprise a disproportionate portion of the world’s poor and experience far worse health outcomes than their non-indigenous counterparts [3]. Malnutrition contributes greatly to these observed health disparities and is a focus of humanitarian support, government intervention, and research in these populations [2]. The dietary crisis within indigenous communities stems from both overall insufficient food levels and a lack of access to nutritious options partly due to insufficient storage methods to ward off contamination [2].

Ten percent of the world’s indigenous population reside in Latin America and the Caribbean, among more than 400 distinct people groups [4]. The Kuna Indians are an indigenous community primarily residing on the San Blas Islands off the east coast of Panamá. The dietary habits of the island Kuna were initially studied in the early 1990s in an attempt to explain the indigenous population’s low levels of age-related hypertension when compared to Panama City residents [5]. The findings of this study suggested major dietary differences among island dwelling Kuna communities compared to non-indigenous Panamanian residents, primarily through the consumption of cacao beverages [5, 6]. It is surmised that the island Kuna have the highest rate of flavonoid consumption globally [6]. This cacao beverage is rich in flavonoids which stimulate nitric oxide production leading to vasodilation and decreased blood pressure [6]. Researchers believed it was this high level of flavonoid consumption and therefore sustained activation of the nitric oxide system that led to the island Kuna’s increased life expectancy, and decreased incidence of common chronic conditions such as hypertension, cardiovascular disease, stroke, diabetes, and cancer [5, 6]. However, after these initial studies there has been a paucity of research completed on food consumption in this population.

While the studies conducted in the island Kuna focused primarily on cacao consumption, other dietary changes have been noted in indigenous communities which may be occurring in the Kuna of Panama [5–10]. For example, the global increase in urbanization among indigenous populations has brought with it a Westernization of indigenous health concerns with dietary and lifestyle changes being major contributors [2]. This includes consumption of more calorically dense foods, increased sodium intake, and lowered nutritional intake of fiber [2, 11]. It is suggested that this dietary transition has increased the prevalence of chronic disease in the indigenous population including obesity, high blood pressure, heart disease, type 2 diabetes, and renal disease [2, 11]. Evidence also suggests that ultra-processed foods are of significant dietary concern for some indigenous communities, such as members of the First Nation communities whose major source of dietary energy comes from processed foods [12].

While some evidence exists on the dietary habits of the Kuna Indians, much of the data is out dated and does not account for dietary transitions that may have taken place in the last decade. For example, the initial dietary surveys were administered in the early 1990s in the Kuna Indian communities and much of the research conducted on dietary habits of the Kuna have focused on the role of cacao. Additionally, information on the current environment, lifestyles, and health outcomes of the Kuna Indians is lacking in the scientific literature, necessitating empirical baseline information on food intake among the Kuna Indians. As many indigenous communities around the world have demonstrated transitions in dietary habits that are leading to poor health [2, 11, 12], assessment of the current dietary trends among the Kuna are highly warranted in order to understand health from a nutritional stand point. Therefore, this paper aims to provide new information on dietary habits and sociodemographic differences that may occur within diet for the Kuna Indian communities living off the coast of Panama.

## Materials and methods

### Data collection

Lay health workers from the community were trained to administer a paper-based survey instrument that was used to collect data. The study questionnaire was developed based on standard validated and available questionnaires. Specifically, measures from the “Panamanian prevalence of risk factors associated with cardiovascular disease in 18 year or older population survey” [13] was included in the study questionnaire. In addition, demographic information was collected that included asking participants to report their age, sex, education, marital status, literacy, and income level.

### Participants and recruitment

**Fig. 1:**
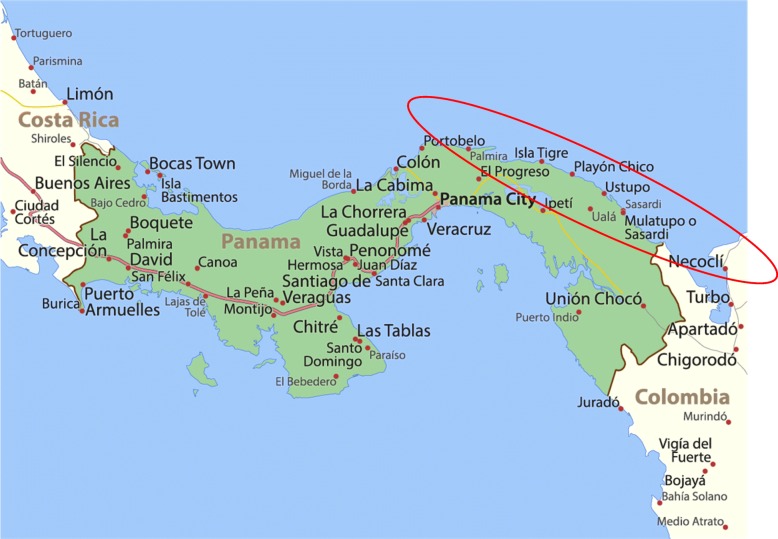
Map of Panama. Red circle indicates the location of the San Blas Islands

This is a cross-sectional study with a convenience sample of 211 adults dwelling in the indigenous zone of Ustupu and Ogobsucum, see Fig. 1 for Map of location. Study recruitment took place in two primary locations, the local clinic and throughout the general community. Clinic recruitment was completed by lay health workers announcing the study to patients in the clinic waiting areas. Community recruitment was completed through community leaders announcing the study at a community meeting. Interested participants were provided a study overview and verbal consent was obtained. After obtaining consent, participants completed the paper-based survey individually or if requested by participants, lay health workers administered the survey in an interview format by reading questions allowed and documenting responses. Overall, each survey took approximately 30 min to 1 h to complete. Participants were given as much time as was necessary to complete the survey. Participation was completely voluntary, and no incentives were provided for completion of this survey. It should be noted that there is no regular census data for Ustupu and Ogobsucum; therefore, a sampling strategy was not developed for recruitment. Response rate for recruitment was high with approximately 90% of participants approached agreeing to participate.

### Outcomes

Nutrition and food groups were evaluated using a standard validated food frequency questionnaire from the “Panamanian prevalence of risk factors associated with cardiovascular disease in 18 year or older population survey” [13]. These included:

“In a normal week how many times do you eat the following foods?”

- Apple or pear; banana; grapes, watermelon, melon; papaya, pineapple; and, orange, lime or mango. Other fruits that weren’t listed but were mentioned by respondents included raisins and dried fruit. These items were categorized as **Fruits**.
- Squash, beans, spinach, broccoli, greens; lettuce, cucumber, cabbage; tomato, carrot. These items were categorized as **Vegetables.**
- Fish that has not been fried, canned tuna. These items were categorized as **Fish.**
- Soda, canned or boxed juice, artificially flavored drinks (i.e., Kool-Aid). These items were categorized as **Soda/Sugary beverages.**
- Tortilla, empanadas; French fries, fried plantain, fried yuca; fried sausage; fried nuggets; fried meats. These items were categorized as **Fried foods**.
- Cacao or dark chocolate. These items were categorized as **Cacao.**
- Chips, Doritos, caramels, chocolate bars. These items were categorized as **Junk food**.
- McDonalds, Pio Pio, Burger King, or other fast food. These items were categorized as **Fast foods.**

The final outcome categories included: fruits, vegetables, fish, soda/sugary beverages, fried foods, cacao, junk food, and fast foods.

Frequency of food group consumption—fruits, vegetables, fish, sodas, fried foods, cacao, and junk food—were categorized for each individual as daily vs. not daily. Responses of every day were categorized as daily, while responses of 3–6 times a week, less than 3 days a week, and never were categorized as not daily. Fast foods were categorized as weekly consumption vs. less than weekly. Responses of every day, 3–6 times a week, and less than 3 times a week were categorized as weekly. The response of ‘never’ was categorized as less than weekly.

### Covariates

Standard demographic information was collected by self-report: Age (as a continuous variable); sex (as a dichotomous male/female); education (primary (0–6 years of school), Secondary (7–12 years of school), Some college (13–17), College graduate or higher (18+)); and monthly family income. For income, respondents were asked to select which of the following best describes their monthly income: (< $250; $250 - $300; $301 - $600; $601 - $999; $1000 - $1200; >$1200; or I don’t know). Income was collected based on monthly numbers after consulting with community leaders on income levels within the context of their community. Based on the community feedback and context, responses to income were categorized as follows: poor income (don’t know,), low-moderate income (less than $250) and moderate-high income ($250 and above). Data on family structure was collected which was defined by the number of individuals supported by the family income. Literacy was assessed by asking whether respondents were able to read and write. Participants were as to defined their marital status as married, single, separated, divorced, widowed, or other union. Marital status was defined as not married (single, separated, divorced, widowed) or married if married or involved in an ‘other union’.

Physical Activity was assessed using two questions. The first question was, “Do you participate in physical activity?” If the individual responded yes, then they were asked to select the activities in which they participated, including walking, swimming, soccer, volleyball, running, track, baseball, cycling, basketball, housework, spinning or stationary biking, weight lifting, carrying water or food, work on the farm (cutting grass, cutting sugar cane), tennis, construction work, lifting weights in the gym, walking to work from your home, other sports, other physical activities, other aerobic activities. The final question was, “How many days per week and for how long do you do your activity?” Participants responded with a number of days from 1 to 7 and the average minutes per day the activity was conducted.

### Statistical analysis

All analysis was run using Stata v.14, with significance determined based on a two tailed alpha of *p* < 0.05. First, frequencies were calculated to describe the demographic factors for the sample population (age, sex, education, monthly income, marital status, number of dependents, and literacy). Secondly, proportions and confidence intervals for prevalence of each of the dietary categories (fruits, vegetables, cacao, fish, sodas, fried food, fast food, and junk food) were calculated and then compared using chi2 tests for categories of demographic factors. Thirdly, adjusted logistic regression models for independent correlates of dietary categories were analyzed. In each model age, gender, education, and income were added as primary independent variables, with separate models run for each dietary category as the outcome.

## Results

Demographic characteristics for the sample population (*N* = 211) are provided in Table 1. The majority of participants were women (73.0%), married (70.4%), and literate (79.3%). Of those completing the survey, 40.1% were between the ages of 18 and 39 years, 17.6% had no education, 37.0% reported having a monthly income below $250 (low-moderate income), and nearly one-third (29.3%) reporting a family structure consisting of 6 to 8 dependents.

**Table 1:** Sample Demographics (*n* = 211)

	Percentage
Age	
18–39	83 (40.1%)
40–59	67 (32.4%)
60–90	57 (27.5%)
Sex	
Male	57 (27.0%)
Female	154 (73%)
Education	
None	35 (17.6%)
Primary	57 (28.6%)
Secondary/University	107 (53.8%)
Monthly Income	
Poor	108 (51.9%)
Low-Moderate	77 (37.0%)
Moderate-High	23 (11.1%)
Married	
No	56 (29.6%)
Yes	133 (70.4%)
Can Read/Write	
No	43 (20.7%)
Yes	165 (79.3%)
Family Dependents	
1 to 5	52 (25.0%)
6 to 8	61 (29.3%)
9 to 11	50 (24.0%)
12 to 22	45 (21.6%)
Regular Physical Activity	189 (89.8%)
Alcohol in past 6 months	117 (55.6%)
Tobacco products in last 30 days	13 (6.2%)

Education – none: < 5th grade; primary: 5th grade - <12th grade; secondary: > = 12th grade

Income – poor: don’t know; low-moderate: < $250; moderate-high: > = $250

**Fig. 2:**
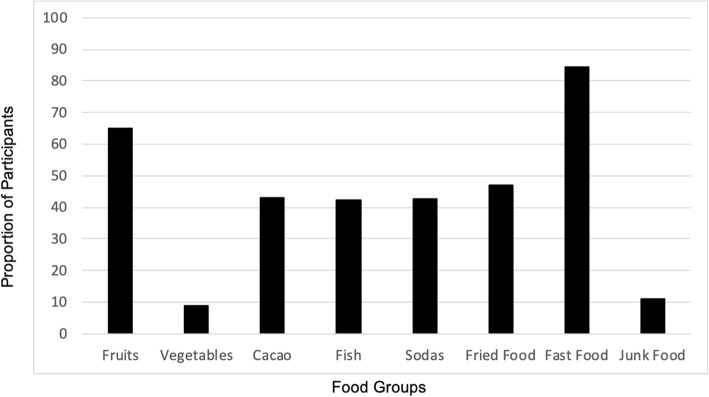
Proportion of Participants Consuming Each Food Group Daily and Fast Food Weekly. Frequency of food group consumption were categorized for each individual as daily vs. not daily. Responses of every day were categorized as daily, while responses of 3–6 times a week, less than 3 days a week, and never were categorized as not daily. Fast foods were categorized as weekly consumption vs. less than weekly. Responses of every day, 3–6 times a week, and less than 3 times a week were categorized as weekly. The response of ‘never’ was categorized as less than weekly

Figure 2 provides proportions of the population reporting specific food consumption. The majority (84.4, 95% CI 78.7–89.0) of respondents reported weekly fast food consumption. On the other hand, reported daily consumption of food groups varied: fruit (64.9, 95% CI, 58.1–71.4), fried food (46.9, 95%CI 40.0–53.9), cacao (42.9, 95% CI 36.1–49.8), soda (42.7, 95% CI 35.9–49.6), fish (42.5, 95% CI 35.7–49.6), junk food (10.9, 95% CI 7.0–15.9), and vegetables (9.0, 95% CI 5.5–13.7).

Table 2 provides results of the unadjusted relationship between demographic characteristics and food type consumption. Significant differences existed between income categories and many of the dietary categories, including cacao (*p* = 0.02), fish (*p* = 0.001), sodas (*p* = 0.02), fast food (*p* < 0.001), and junk food (*p* = 0.04). Significant differences also existed for fast food consumption by levels of education (*p* < 0.001), sex (*p* = 0.001), and age (*p* = 0.001). Finally, significant differences existed between age categories for vegetable consumption (*p* = 0.03).

**Table 2:** Unadjusted Relationships to Understand Differences in Food Consumption by Socio-demographic Variables

	Fruits (95% CI)	Vegetables (95% CI)	Cacao (95% CI)	Fish (95% CI)	Sodas (95% CI)	Fried Food (95% CI)	Fast Food (95% CI)	Junk Food (95% CI)
Age	*p = 0.23*	***p = 0.03***	*p = 0.99*	*p = 0.12*	*p = 0.05*	*p = 0.16*	***p = 0.001***	*p = 0.50*
18–39	57.8% (46.5–68.6)	7.2% (2.7–15.1)*	42.7% (31.8–54.1)	40.2% (29.6–51.7)	38.6% (28.1–49.9)	48.2% (37.1–59.4)	73.5% (62.7–82.6)**	13.3% (6.8–22.5)
40–59	70.2% (57.7–80.7)	16.4% (8.5–27.5)*	43.3% (31.2–56)	35.4% (23.9–48.2)	35.8% (24.5–48.5)	53.7% (41.1–50.7)	86.6% (76.0–93.7)**	11.9% (5.3–22.2)
60–90	68.4% (54.8–80.1)	3.5% (0.4–12.1)*	43.9% (30.7–57.6)	53.6% (39.7–67)	56.1% (42.4–69.3)	36.8% (24.4–50.7)	96.5% (87.9–99.6)**	7.0% (1.9–17.0)
Sex	*p = 0.52*	*p = 0.94*	*p = 0.09*	*p = 0.70*	*p = 0.60*	*p = 0.82*	***p = 0.001***	*p = 0.70*
Male	68.4% (54.8–80.1)	8.8% (2.9–19.3)	33.3% (21.4–47.1)	40.4% (27.6–54.2)	45.6% (32.4–59.3)	45.6% (32.3–59.3)	70.2% (56.6–81.6)**	12.3% (5.1–23.7)
Female	63.6% (55.5–71.2)	9.1% (5.1–14.8)	46.4% (38.3–54.6)	43.3% (35.3–51.7)	41.6% (33.7–49.8)	47.4% (39.3–55.6)	89.6% (83.7–93.9)**	10.4% (6.1–16.3)
Education	*p = 0.20*	*p = 0.86*	*p = 0.71*	*p = 0.17*	*p = 0.72*	*p = 0.99*	***p < 0.001***	*p = 0.47*
None	80.0% (63.1–91.6)	11.4% (3.2–26.7)	48.6% (31.4–66.0)	45.5% (28.1–63.6)	45.7% (28.8–63.4)	45.7% (28.8–63.4)	100.0% (90–100)***	5.7% (0.7–19.2)
Primary	66.7% (52.9–78.6)	8.8% (2.9–19.3)	40.4% (27.6–54.2)	48.2% (34.7–62)	42.1% (29.1–55.9)	47.4% (34–61)	96.5% (87.9–99.6)***	14.0% (6.3–25.8)
Secondary	63.6% (53.7–72.6)	8.4% (3.9–15.4)	41.5% (32–51.5)	34.0% (25–43.8)	38.3% (29.1–48.2)	46.7% (37–56.6)	71.0% (61.5–79.4)***	11.2% (5.9–18.8)
Monthly Income	*p = 0.05*	*p = 0.22*	***p = 0.02***	***p = 0.001***	***p = 0.02***	*p = 0.52*	***p < 0.001***	***p = 0.04***
Poor	72.2% (62.8–80.4)	6.5% (2.6–12.9)	51.4% (41.5–61.2)*	54.8% (44.7–64.6)**	50.9% (41.1–60.7)*	50.0% (40.2–59.8)	95.4% (89.5–98.5)***	15.7% (9.4–24)*
Low-Moderate	57.1% (45.4–68.4)	7.8% (2.9–16.2)	37.7% (26.9–49.4)*	28.6% (18.8–40)**	29.9% (20–41.4)*	41.6% (30.4–53.4)	70.1% (58.6–80)***	5.2% (1.4–12.8)*
Moderate-High	52.2% (30.6–73.2)	17.4% (5–38.8)	21.7% (7.5–43.7)*	30.4%(13.2–52.9)**	47.8% (26.8–69.4)*	47.8% (26.8–69.4)	78.3% (56.3–92.5)***	4.4% (0.01–21.9)*

*p < 0.05, **p < 0.01, ***p < 0.001

Table 3 presents results of the adjusted logistic regression for each dietary category. A similar trend emerges with monthly income significantly independently associated with a number of the dietary categories: fruits, cacao, fish, sodas, fast food, and junk food. Both low-moderate and moderate-high groups were less likely to consume fruits (low-moderate OR = 0.38, 95% CI 0.19, 0.78; moderate-high OR = 0.28, 95% CI 0.10, 0.75) and fast food (low-moderate OR = 0.15, 95% CI 0.04, 0.46; moderate-high OR = 0.31, 95% CI 0.06, 1.42) compared to poor individuals. Moderate-high income individuals were less likely than poor individuals to consume cacao (OR = 0.27, 95% CI 0.09, 0.81). Low-moderate income individuals were less likely to consume fish (OR = 0.28, 95% CI 0.13, 0.58), sodas (OR = 0.35, 95% CI 0.17, 0.73), and junk food (OR = 0.21, 95% CI 0.06, 0.75) compared to poor individuals. In addition to income, those age 60–90 were more likely than those age 18–39 to consume fast food (OR = 12.17, 95% CI 2.00, 73.84); women were more likely than men to consume fast food (OR = 3.43, 95% CI 1.23, 9.56), and those with a primary education were more likely than those with no education to consume fast food (OR = 4.83, 95% CI 1.01, 23.0) Finally, there was a significant independent association between the oldest age group consuming more fish and soda compared to the youngest age group (fish OR for age 60–90 = 3.24, 95% CI 1.11, 9.44; soda OR for age 60–90 = 3.90, 95% CI 1.38, 11.04).

**Table 3:** Adjusted Logistic Regression for Independent Correlates of Dietary Habits (OR, 95%CI)

	Fruits	Vegetables	Cacao	Fish	Sodas	Fried Food	Fast Food	Junk Food
Age								
18–39 (ref)	–	–	–	–	–	–	–	–
40–59	1.45 (0.68, 3.08)	2.25 (0.69, 7.98)	1.09 (0.53, 2.24)	1.04 (0.48, 2.24)	1.35 (0.64, 2.85)	1.29 (0.64, 2.60)	2.77 0.96, 7.98)	1.10 (0.36, 3.19)
60–90	1.16 (0.40, 3.35)	0.43 (0.06, 3.26)	1.33 (0.48, 3.66)	**3.24 (1.11, 9.44)**	**3.90 (1.38, 11.04)**	0.51 (0.19, 1.40)	**12.17 (2.00, 73.84)**	0.58 (0.12, 3.07)
Sex								
Male (ref)	–	–	–	–	–	–	–	–
Female	0.51 (0.22, 1.19)	0.69 (0.18, 2.66)	1.26 (0.56, 2.83)	1.20 (0.49, 2.90)	1.07 (0.46, 2.48)	0.79 (0.36, 1.73)	**3.43 (1.23, 9.56)**	0.41 (0.11, 1.55)
Education								
None (ref)	–	–	–	–	–	–	–	–
Primary	0.44 (0.13, 1.51)	0.74 (0.11, 5.09)	0.82 (0.28, 2.40)	2.28 (0.71, 7.26)	1.44 (0.48, 4.28)	0.76 (0.26, 2.23)	**4.83 (1.01, 23.0)**	1.82 (0.27, 12.20)
Secondary	0.43 (0.11, 1.67)	0.63 (0.08, 4.93)	0.99 (0.30, 3.26)	1.80 (0.50, 6.38)	1.87 (0.57, 6.20)	0.66 (0.20, 2.15)	–	1.37 (0.17, 10.84)
Monthly Income								
Poor (ref)	–	–	–	–	–	–	–	–
Low-Moderate	**0.38 (0.19, 0.78)**	1.28 (0.35, 4.62)	0.58 (0.30, 1.14)	**0.28 (0.13, 0.58)**	**0.35 (0.17, 0.73)**	0.61 (0.31, 1.19)	**0.15 (0.04, 0.46)**	**0.21 (0.06, 0.74)**
Moderate-High	**0.28 (0.10, 0.75)**	3.17 (0.72, 13.96)	**0.27 (0.09, 0.81)**	0.39 (0.13, 1.11)	0.97 (0.37, 2.58)	0.84 (0.32, 2.17)	**0.31 (0.06, 1.42)**	0.16 (0.02, 1.39)

Bold indicates significance at *p* < 0.05 based on logistic regression

Model is adjusted for sex, age, education, and monthly income

*CI* Confidence interval

## Discussion

Among this sample of Kuna Indian adults, 85% reported eating fast food at least one time weekly, 47% reported eating fried food daily, and 11% reported eating junk food daily. After adjusting for covariates, women reported eating more fast food than men and the 60–90-year old age group ate fast food more times per week than those 18–39 years. Those with a primary level of education at fast food more often than those with no education; however, those with a higher income were less likely to eat fast food weekly than those in the lowest income category. Respondents with low-moderate income levels ate junk food and sodas less often; however, they also ate fish and fruits less frequently than those categorized as poor.

To our knowledge this is the first dietary survey study of the island Kuna that focuses on food groups outside of cacao [5–10]. Though, Hollenberg and colleagues (1997) found that Kuna Indians have substantial sodium intake, where 75% of intake was due to table salt, but fat and protein intake were low in comparison to US dietary patterns [5]. Given the growing concern of cardiometabolic disease in lower income populations, and the changes seen in diet in other indigenous groups globally, these findings provide important information to guide efforts targeted at the health of Kuna Indians [14]. A study in Botswana found that while the traditional indigenous foods were recognized as healthful options, women reported a decrease in consumption due to favorability of western [15]. Prior survey research of indigenous dietary patterns has been conducted with similar results indicating high prevalence of fast food or processed foods [12]. In one such study, researchers found ultra-processed foods to represent 53.9% of Canadian First Nation energy consumption in participants aged 19 and older [12]. This increase in Western food resulted in higher carbohydrate, saturated fat and sodium and less protein and fiber than the traditional non-ultra processed diet [12]. The increased reliance on processed foods may be due to the limited availability of designated cultivable land or an increase in reliance on storable Western foods introduced through economic and government shifts as a result of globalization [16]. Furthermore, a published review of research on the rise in ultra-processed foods in lower-income countries proposed the production and marketing influence of western fast food companies to be major factors that influence indigenous dietary patterns [17].

In the current study sample, income was noted as an important correlate of dietary patterns. Participants reporting an unknown income were described by the community as the lowest income group, given they do not have a regular monthly income on which to live and all income is spent on survival with little to no money coming back to the home in order to estimate monthly income. Particularly in this Kuna population, which resides on islands off the coast of Panama, communities have limited access to agricultural land. However, those with higher income levels were less likely to eat fish or fruits daily, compared to those who were poor. This may be indicative of the traditional reliance on fish as a staple diet item and the higher availability of fruit compared to vegetables in the community. While the increased rate of unhealthy food choices in the lowest income group is cause for concern, the continued traditional protein source of fish is cause for optimism. It is noted that fish consumption may decrease risk of cardiovascular disease and the American Heart Association recommends at least two servings of fish per week. As such, fish consumption could be used as a focus for community interventions [18]. In addition, given the very low percentage of individuals consuming vegetables daily, it may also be important to increase vegetable consumption throughout the community through increase agricultural production.

Strengths of this study include self-reported information from a difficult to reach and rarely accessed indigenous group in Latin America. However, limitations exist, including the cross-sectional nature of the data which cannot speak to trends in food consumption in the population, and the inability to generalize beyond the confines of the Kuna Indians. An additional limitation is the food intake questionnaire used for this study. Though this survey was deemed the most appropriate to capture the data, it is broad and does not cover all food categories. For example, there may be particular food types more commonly accessible on the island, or specific vegetables that are not common in Panama but used on the island. Collection of qualitative data in the future to improve the food frequency questionnaire may be useful for ongoing studies. It should also be noted that there is limited data and literature to help explain why there is an increased uptake of Western foods among Kuna Indians. However, the current study has provided a comprehensive assessment of food intake that will contribute to the Kuna-specific literature. Lastly, as no census information exists for the population a detailed sampling frame could not be determined. The convenience sample is one reason there may be an overrepresentation of women in this study population, particularly given men often leave the islands for periods of time to work on the mainland. Future work should specifically target men to increase recruitment. Still, the results are strengthened by the decade long relationship and trust building between researchers and the community, which increased the response to completion of the survey. This relationship will provide the opportunity for continued collaboration in addressing community health concerns.

## Conclusion

In conclusion, little work has been conducted on developing culturally appropriate nutrition focused programs for indigenous communities. This study found that the Kuna Indians are consuming a diet that has integrated foods considered to be unhealthy, such as fast food, junk food, and soda, into their diet. In addition, there was a relationship between food types and sociodemographic factors, with low income populations consuming unhealthy food types more frequently. Findings from this study suggest that the community could benefit from interventions to attain affordable and healthy alternatives. Further research is needed to develop culturally relevant nutrition programs, particularly with the lowest income subset of the island Kuna population.

## Data Availability

The dataset generated and analyzed during the current study is not available because of confidentiality agreement with the study communities.

## References

[1] Bartlett JG, Madariaga-Vignudo L, O’Neil JD, Kuhnlein HV. Identifying indigenous peoples for health research in a global context. Int J Circumpolar Health. 2007;66(4):287–307. 10.3402/ijch.v66i4.18270 18018843

[2] Gracey M, King M (2009). Indigenous health part 1: determinants and disease patterns. Lancet..

[3] Stephens C, Nettleton C, Porter J, Willis R, Clark S (2005). Indigenous peoples’ health--why are they behind everyone, everywhere?. Lancet..

[4] Montenegro RA, Stephens C (2006). Indigenous health in Latin America and the Caribbean. Lancet..

[5] Hollenberg NK, Martinez G, McCullough M (1997). Aging, acculturation, salt intake, and hypertension in the Kuna of Panama. Hypertension..

[6] Bayard V, Chamorro F, Motta J, Hollenberg NK (2007). Does flavanol intake influence mortality from nitric oxide-dependent processes? Ischemic heart disease, stroke, diabetes mellitus, and cancer in Panama. Int J Med Sci.

[7] Hollenberg NK, Fisher ND, McCullough ML (2009). Flavanols, the Kuna, cocoa consumption, and nitric oxide. J Am Soc Hypertens.

[8] Hollenberg NK, Mohres E, Meinking T (2005). Stress and blood pressure in Kuna Amerinds. J Clin Hypertens (Greenwich).

[9] Hollenberg NK, Rivera A, Meinking T (1999). Age, renal perfusion and function in island-dwelling indigenous Kuna Amerinds of Panama. Nephron..

[10] Hollenberg KN (2006). Vascular action of cocoa flavanols in humans: the roots of the story. J Cardiovasc Pharmacol.

[11] Whiting SJ, Mackenzie ML (1998). Assessing the changing diet of indigenous peoples. Nutr Rev.

[12] Batal M, Johnson-Down L, Moubarac JC (2018). Quantifying associations of the dietary share of ultra-processed foods with overall diet quality in first nations peoples in the Canadian provinces of British Columbia, Alberta, Manitoba and Ontario. Public Health Nutr.

[13] McDonald A, Motta J, Roa R, Batista I, Correa R, Gonzalez B. Prevalencia de factores de riesgo asociados a enfermedad cardiovascular en la población adulta de 18 años y más. Provincias de Panamá y Colón: Ministerio de Salud. Panama City: Ministerio de Salud - Panama; 2011.

[14] Bollyky TJ, Templin T, Cohen M, Dieleman JL (2017). Lower-income countries that face the Most rapid shift in noncommunicable disease burden are also the least prepared.

[15] Kasimba SN, Motswagole BS, Covic NM, Claasen N (2018). Household access to traditional and indigenous foods positively associated with food security and dietary diversity in Botswana. Public Health Nutr.

[16] D'Ambrosio U, Puri RK (2016). Foodways in transition: food plants, diet and local perceptions of change in a Costa Rican Ngabe community. J Ethnobiol Ethnomed.

[17] Monteiro CA, Moubarac JC, Cannon G, Ng SW, Popkin B (2013). Ultra-processed products are becoming dominant in the global food system. Obes Rev.

[18] Raatz SK, Silverstein JT, Jahns L, Picklo MJ (2013). Issues of fish consumption for cardiovascular disease risk reduction. Nutrients..

